# Effects of dietary *Lycium ruthenicum* (black goji berry) branches supplementation on growth performance, meat quality, and muscle amino acid and fatty acid composition in sheep

**DOI:** 10.3389/fvets.2025.1616612

**Published:** 2025-07-30

**Authors:** Yan Ma, Yuxia Yang, Liangzhong Hou, Jinlong Li, Pingping Duan, Tongjun Guo

**Affiliations:** ^1^Feed Research Institute, Xinjiang Academy of Animal Sciences, Urumqi, China; ^2^Xinjiang Key Laboratory of Herbivorous Livestock Feed Biotechnology, Urumqi, China

**Keywords:** *Lycium barbarum* (black goji berry), meat quality, amino acids, fatty acid composition, sheep

## Abstract

**Introduction:**

The aim of this study was to investigate the effect of a preparation of black goji berry branches (*Lycium ruthenicum*) on growth performance, meat quality, amino acid and fatty acid content of sheep.

**Methods:**

The experiment was a one-way completely randomized trial, in which 40 male sheep of the F1 generation of Dupo × Lake sheep crosses at four months of age were randomly divided into four groups of ten lambs each. Each group was fed an isoenergetic and isonitrogenous total mixed pellet ration containing 0% (CON), 10% (H1), 20% (H2) and 30% (H3) of *Lycium ruthenicum* branches. The experimental period included a pre-test adaptation of 10 d and an experimental test period of 60 d.

**Results:**

The Luminosity of each experimental group was highly significantly higher than that of the CON (*p* < 0.01). The values for Redness of the H2 and H3 groups were significantly higher than that of the CON (*p* < 0.05). The gamma-aminobutyric acid (GABA) concentration in the longest back muscle first increased and then decreased (*p* < 0.01), and the H2 group was extremely significantly higher than the CON and the other experimental groups. The H1 and H2 groups were significantly lower than the control group, decreasing by 6.87% and 7.07%, respectively (*p* < 0.05). The c20:0, c20:1 content showed a linear increase with increasing addition of *Lycium ruthenicum,* but the difference was not significant (*p* > 0.05).

**Discussion:**

Thus, dietary feed supplementation with 20% of dried, ground *Lycium ruthenicum* branches can improve sheep meat quality and culture benefit.

## 1 Introduction

The rapid development of animal farming has led to insufficient high-fiber forage resources and shortages of grass for animals. This scarcity together with the dependence on only a few types of forage have become more serious and have begun to limit the development of animal husbandry (1). One solution of this problem is to test non-conventional types of roughage as replacements in conventional feeds (2). Increasing the proportion of ground-sourced, non-conventional roughage in the feed can alleviate the resource shortage and reduce feed costs.

*Lycium ruthenicum (L. ruthenicum)* is a deciduous shrub in the *Solanaceae* family, valuable as a food plant but also medicinally useful because of its antioxidant (3), antitumor, lipid-lowering (4), and immune-enhancing properties. Its by-products (stalks, branches, leaves, leftover fruit, etc.) contain beneficial compounds like proanthocyanidins, flavonoids, polysaccharides, alkaloids, amino acids, betaine and other active ingredients (5). These compounds make *L. ruthenicum* by-products promising candidates for use as functional feed additives with bioactive properties (6). *L. ruthenicum* leaf flavonoids inhibit the proteolysis of minced meat and enhance its gelatinous and chewy properties by delaying muscle pH changes, lowering fat peroxide value (POV) and total volatile base nitrogen (TVB-N) content, and altering pancreatic lipase secondary structure by non-covalent reversible binding to trypsin, thereby reducing fat synthesis (7, 8).

Adding a mixture of wolfberry leaves and astragalus to the diets of pigs increased the content of unsaturated fatty acids and vitamin E in pork, which in turn improved meat quality and nutritional value (9). A study by Zhang et al. (10) found that wolfberry by-products and their fermentation products could significantly improve the fatty acid and organic acid contents of lamb by regulating valine, leucine, and isoleucine biosynthesis, linoleic acid metabolism, and glycerophospholipid metabolic pathways, which could affect meat quality. Hou et al. (11) found that the addition of 5% black wolfberry residual fruits to replace part of the roughage significantly increased the growth performance and improved rumen fermentation parameters and gross profit of Dolan sheep.

In order to improve the yield of *Lycium barbarum*, *Lycium barbarum* trees need to be artificially shaped and pruned, producing a large number of *Lycium barbarum* branches. The hypothesis of this paper is that the applicantion of *L. ruthenicum* branches and leaves as livestock feed in animal husbandry can effectively alleviate the problem of insufficient sources of forage, reduce the cost of animal husbandry, improve the production performance of livestock, and improve the economic benefits of farming.

Therefore, this study investigated how different amounts of *L. ruthenicum* by-products added to sheep feed affect carcass characteristics, meat quality, amino acid and fatty acid composition. The goal was to provide scientific evidence for the application of *L. ruthenicum* branches to improve production in an actual sheep farm.

## 2 Materials and methods

### 2.1 Experimental location and ethical statement

The experiments were conducted from Jun 9 to Aug2, 2023 at the sheep farm of Xinjiang Taihe Agriculture and Animal Husbandry Technology Co., Ltd., Bachu County, Kashgar Prefecture, Xinjiang (Kashgar, China). The study was carried out in accordance with the procedures sanctioned for this research, which were approved by the Science and Technology Ethics Committee of Xinjiang Academy of Animal Sciences, China (Approval No. 20230508). These procedures adhere to the principles and regulations for ethical protection in human and animal biological science and technology in China.

### 2.2 Experimental materials

**Figure 1:**
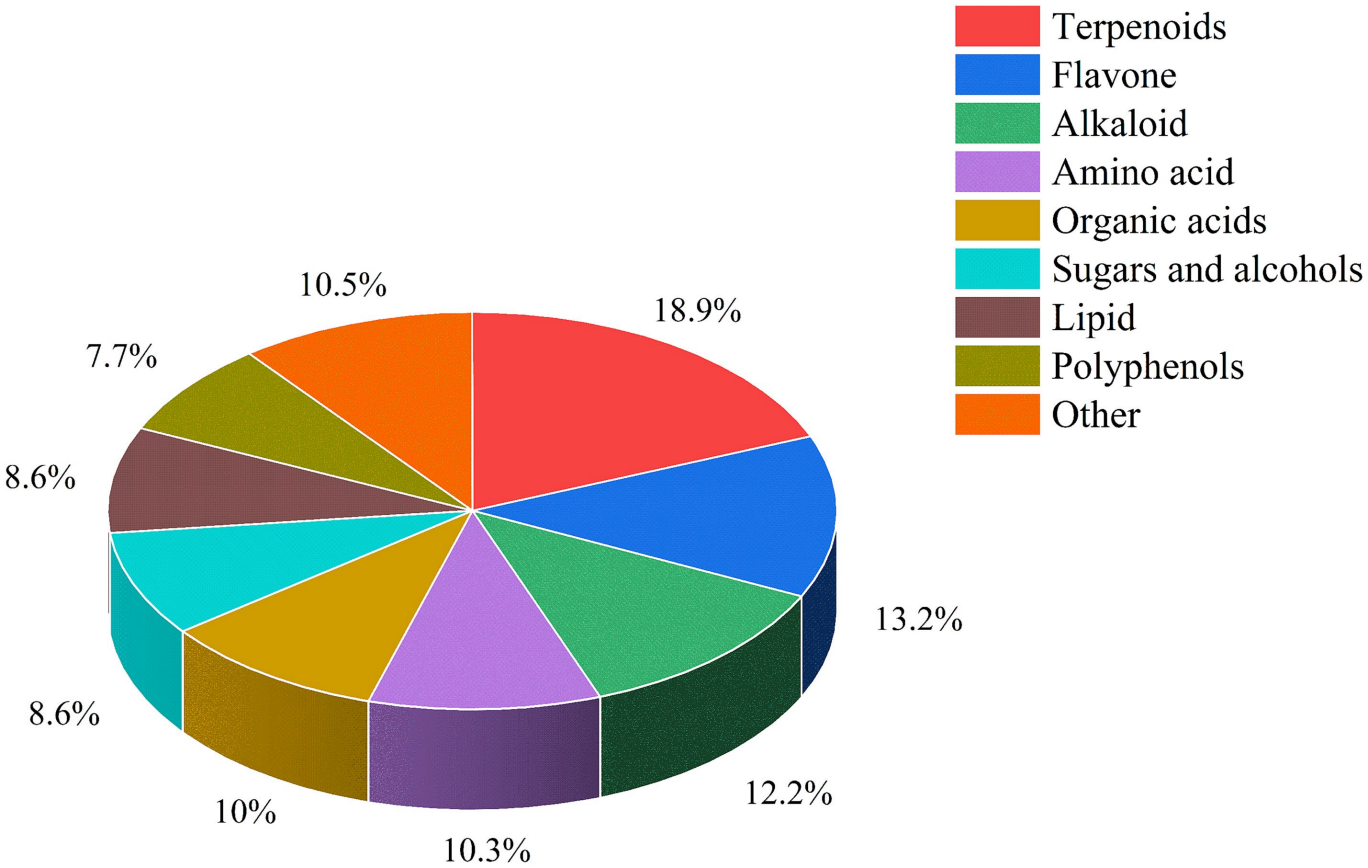
Classification of bioactive substances in *L. ruthenicum* branches.

Air-dried *L. ruthenicum* branches remaining after picking fresh fruit were provided by Xinjiang black fruit goji berry Biotechnology Company, Ltd., and their nutrient composition is shown in Table 1. The detection and quantitation of the bioactive ingredients in *L. ruthenicum* branches was performed by the Baimaike Biological Company, Ltd., using liquid chromatography-mass spectrometry (LC–MS), and the results are shown in Figure 1. The main active components included terpenoids, flavonoids, alkaloids, amino acids, organic acids, sugars, alcohols, lipids and polyphenols. The top five most abundant compounds were terpenoids (18.90%), flavonoids (13.20%), alkaloids (12.20%), organic acids (10.00%), and amino acids (10.30%).

**Table 1:** Nutrient content of *L. ruthenicum* branches as % dry matter (DM).

Item	DM	CP	EE	Ash	NDF	ADF	Ca	P
*L. ruthenicum* branches	94.9	6.15	18.4	8.8	50.3	33.5	12.0	0.14

This study employed a completely randomized design to investigate the effects of incorporating *L. ruthenicum* into the diet of sheep. Forty male sheep (4 months old, 30 ± 2 kg body weight) were dewormed and randomly assigned to four treatment groups (*n* = 10 sheep per group),they are CON, H1, H2, H3 groups. The control group (CON) received the standard diet, while groups H1, H2 and H3 received the same diet supplemented with 10, 20 and 30% *L. ruthenicum* branch roughage [referring to Hou et al. and Duan et al. with minor modifications (11, 12)], respectively (see Table 2 for the composition and nutrient analysis of the diet). The 70-day experimental period included a ten-day acclimation period followed by a 60-day feeding trial. Bedding material selection straw, hay, etc. initial laying thickness 15–20 cm, subsequent weekly replenishment. Adjusted according to humidity, usually 1–2 weeks partial replacement, once a month thoroughly cleaned. The temperature inside the sheep house is 10–24°C. A ridged roof with adjustable skylights is used. The eaves are 3–4 meters high to promote air convection and relative humidity is maintained at 60–70%.

**Table 2:** Percent composition and nutrient levels of the basal diet (DM basis).

Items	Groups			
	CON	H1	H2	H3
Ingredients (%)				
Alfalfa hay	10.88	7.54	5.02	3.66
Soybean stalks	14.03	11.12	7.02	5.05
Straw	18.12	14.49	11.9	5.2
*L. ruthenicum* branch roughage	0	10	20	30
Corn	31.04	31.1	30.48	30.36
Wheat bran	7.89	7.74	7.67	7.72
Cottonseed meal	8.88	8.92	8.85	8.89
Sunflower seed meal	4.98	4.92	4.89	4.93
Limestone	0.12	0.12	0.11	0.13
NaCl	0.53	0.53	0.53	0.53
CaHPO_4_	0.51	0.5	0.51	0.51
NaHCO_3_	0.51	0.51	0.51	0.51
Premix^1^	2.51	2.51	2.51	2.51
Total	100.00	100.00	100.00	100.00
Nutritional level^2^				
ME, MJ/kg	11.06	11.21	11.69	11.66
CP, %	13.96	13.99	13.98	13.99
NDF, %	31.12	33.54	33.46	36.84
ADF, %	16.8	18.74	19.31	21.64
Ca, %	0.695	0.625	0.897	0.833
P, %	0.485	0.098	0.44	0.395

^1^The premix provided the following per kg of the diet: VA 150,000 IU, VD3 56,500 IU, VE 8,000 IU, Se (as sodium selenite) 14 mg, I (as potassium iodide) 58 mg, Cu (as copper sulfate) 290 mg, Mn (as manganese sulfate) 1,925 mg, Zn (as zinc oxide) 2,050 mg, Co (as cobalt sulfate) 24 mg.

^2^Calculated values.

### 2.3 Experimental design and animal feeding

Before the test, the sheep pens were cleaned and disinfected, and the test sheep were sheared, dewormed, and bathed. During the test period, the sheep were kept in separate pens and fed twice a day, at 10:00 a.m. and 18:00 p.m. The pens were equipped with a single water trough and a feed trough, and the sheep were free to feed and drink during the period.

### 2.4 Sample collection

#### 2.4.1 Growth

Each sheep was weighed on an empty stomach before morning feeding on d 0 and d 60 (9.00 AM) of the positive trial period, and the amount of feed and leftover material was recorded. Initial body weight, final body weight, average daily gain (ADG), average daily feed intake (ADFI) and feed/gain (F/G) ratio were calculated. According to the following formulas:

Total weight gain(kg)=final weight(kg)−initial weight(kg)

$\mathrm{ADG}\;(\mathrm{kg}/d)=(\begin{array}{l} \text{final weight of}\\ \text{each sheep}-\\ \text{initial weight of}\\ \text{each sheep} \end{array})/\text{experimental}\;\mathrm{day};$

F/G=DMI/ADG .

#### 2.4.2 Carcass traits

The live weight (kg) of each experimental sheep was measured and recorded just before slaughter. The test sheep were bled through the carotid jugular vein, and the head, hoof, skin, and viscera of the test sheep were removed, while the kidneys were retained. The carcass weight was measured and recorded immediately after slaughter, and slaughter percentage was calculated as carcass wgt/live wgt × 100. A longitudinal incision was made on the longissimus dorsi (LD) muscle between the 12th and 13th ribs to obtain the area of the eye muscle. A Vernier caliper was used to measure the length of the longest and widest parts of the eye muscle. The eye muscle area (cm2) was calculated as the longest value of the eye muscle cross section (cm) × the widest value (cm) × 0.7 (13).

#### 2.4.3 Meat quality

Meat quality measurements included physical indicators (pH, color, water loss and cooking loss) and chemical indicators (water, protein, fat and ash contents). Muscle pH was measured using a calibrated portable muscle tissue pH meter (HI99163, Shanghai Jiahui Instrumentation Co.). Meat color indices were measured in the dark 45 min after slaughter using a carcass meat color meter (Meat-5; Beijing Tianxiang Feiyu Technology Co.) and the determinations included L* (brightness), a* (redness), and b* (yellowness). A 1-cm-thick sample was cut vertically along the longest muscle fiber of the back using a circular sampling tool with a diameter of 3 cm (DL-100; Shandong Shunsheng Zhongshi, Shandong, China). To measure the loss in weight from cooking, a 3 × 3 × 5-cm sample of meat was dried with a paper towel, weighed (W1, g), placed into a bag, sealed, and heated in a water bath at 85°C for 40 min. After cooling to room temperature, it was patted dry with a paper towel and then weighed (W2, g). Cooking loss was calculated by subtracting W2 from W1. AOAC (14) methods were used to quantitate the moisture content (Method 950.46), crude protein (Method 928.08), crude fat (Method 960.39), and ash (Method 920.153) in the meat.

#### 2.4.4 Amino acids

For determination of amino acid composition and concentrations, 100 mg samples of lyophilized tissues were homogenized in 1.2 mL of 10% sulfosalicylic acid, then centrifuged at 13,500 × g for 15 min at 4°C. Supernatants were removed and filtered through a 0.22 μm membrane filter into a 2.0 mL glass vial, and amino acid composition and concentration were determined with a high-speed amino acid analyzer (L-8900, Hitachi High-Tech Corporation, TKY, Japan).

#### 2.4.5 Fatty acids

Total fatty acids (TFAs) in frozen meat samples were extracted according to the method of Liang et al. (15). The fatty acids (FAs) were separated by gas chromatography (GC-450, Varian Co., Walnut Creek, CA, USA), and the sample peaks were identified by retention time. The concentrations of individual FAs were determined using standard curves generated from known standard compounds (mixture of C4-C24 methyl esters; Sigma-Aldrich, Inc., St. Louis, MO, USA).

### 2.5 Statistical analysis of data

Data were organized using Excel 2016, and one-way analysis of variance (ANOVA) was performed using the ANOVA procedure of SPSS 26.0 statistical software. Multiple comparisons were performed using Duncan’s method if the differences were significant. The results were expressed as the mean and standard error of the mean (SEM) and regression analysis was done to test for linearity of the effects of increasing amounts of *L. ruthenicum* branches added to feed. The differences were taken as significant at the *p* < 0.05 level and highly significant at *p* < 0.01. Among them, the overall *p*-value refers to testing whether there is a significant difference between the means of all groups to determine the effect of the overall effect on the corresponding detection indexes with the increase in the addition of *L. ruthenicum* branches; the linear *p*-value refers to testing whether the corresponding detection indexes show a linear trend with the level of *L. ruthenicum* branches added when the amount of *L. ruthenicum* branches added increases; and the quadratic *p*-value refers to whether the corresponding detection indexes show a nonlinear (quadratic) trend.

Statistical analysis was performed using bivariate correlation analysis in SPSS 26.0 statistical software, the Pearson algorithm was used to calculate the correlation coefficients, and heat maps of the correlation were created using the heat map tool in Hiplot Pro (https://hiplot.com.cn/). Red color indicates a positive correlation, and blue color indicates a negative correlation. The color shade indicates the magnitude of the correlation. * represents 0.01 < *p* ≤ 0.05, and ** represents *p* ≤ 0.01.

## 3 Results

### 3.1 Growth performance

As can be seen in Table 3, the final weight, ADG of the sheep increased linearly with increasing amounts of *L. ruthenicum* branches added to the feed (*p* = 0.007); the H3 group was significantly higher than the CON, with an increase of 9.89, 41.34% (*p <* 0.05).

**Table 3:** Effect of different *L. ruthenicum* branches levels on growth performance of sheep.

Item	Groups				SEM	*p*-value		
	CON	H1	H2	H3		Total	Linear	Twice
Initial body weight, kg	29.79	29.48	29.67	29.59	0.277	0.984	0.773	0.854
Final body weight, kg	39.80^b^	41.37^ab^	42.10^ab^	43.74^a^	0.489	0.028	0.007	0.179
Total weight gain, kg	11.91	11.89	12.43	14.15	0.533	0.172	0.065	0.225
ADG, g	166.83^b^	198.13^ab^	207.22^ab^	235.83^a^	0.092	0.050	0.011	0.278
ADFI, kg	1.78	1.69	1.78	1.82	0.035	0.624	0.995	0.207
F/G	10.99	9.33	8.82	8.12	0.556	0.063	0.008	0.785

Differences in superscripts indicate significant differences. CON: Control group; H1:10% *L. ruthenicum* branches; H2:20% *L. ruthenicum* branches; H3: =30% *L. ruthenicum* branches.

### 3.2 Carcass traits

As shown in Table 4, the eye muscle area of H1, H2, H3 groups was significantly higher (*p* < 0.05) than that of the CON group, with increases of 24.35, 22.95, 23.23%, respectively.

**Table 4:** Effect of different *L. ruthenicum* branches levels on carcass traits of sheep.

Items	Groups				SEM	*p*-value		
	CON	H1	H2	H3		Total	Linear	Twice
Carcass weight (kg)	21.4	22.14	24.44	21.4	0.733	0.512	0.253	0.807
Slaughter rate (%)	0.48	0.48	0.47	0.47	0.013	0.996	0.864	0.877
Eye muscle area (cm^2^)	14.25^b^	17.72^a^	17.52^a^	17.56^a^	0.476	0.011	0.002	0.367

For the same row of data, there is no significant difference between groups without letters or with the same letters (*p* > 0.05).

### 3.3 Meat quality

As can be seen in Table 5, supplementation with *L. ruthenicum* branches resulted in L* and a* values that were linearly highly significantly or significantly higher (*p* < 0.01or *p* < 0.05). The L* of H1, H2, H3 groups was highly significantly higher than that of the CON group, with increases of 24.04, 29.51, and 34.38%, respectively (*p* < 0.01). The values for a* of the H2 and H3 groups were significantly higher than that of the CON group by 12.55, 15.35% (*p* < 0.05).

**Table 5:** Effect of different *L. ruthenicum* branches levels on meat quality of sheep.

Items	Groups				SEM	*P*-value		
	CON	H1	H2	H3		Total	Linear	Twice
pH_45min_	6.89	6.86	6.97	6.73	0.072	0.730	0.578	0.516
Protein, %	74.14	74.17	73.51	73.67	0.294	0.836	0.472	0.916
Lipid, %	2.69	2.46	2.23	3.29	0.1667	0.126	0.263	0.051
Cooking loss, %	0.22	0.24	0.23	0.24	0.005	0.769	0.552	0.535
Moisture, %	74.14	74.17	73.51	73.68	0.294	0.836	0.472	0.916
Luminosity, L*	35.28^Bb^	43.76^ABa^	45.69^Aa^	47.41^Aa^	1.462	0.006	0.001	0.139
Redness, a*	10.36^b^	10.25^b^	11.66^a^	11.95^a^	0.279	0.049	0.013	0.675
Yellowness, b*	10.00	10.72	11.69	12.83	0.706	0.555	0.163	0.885

For the same row of data, there is no significant difference between groups without letters or with the same letters (*p* > 0.05), while different uppercase/lowercase letters indicate extremely significant/significant differences (*p* < 0.01/*p* < 0.05). CON: Control group; H1:10% *L. ruthenicum* branches; H2:20% *L. ruthenicum* branches; H3: =30% *L. ruthenicum* branches.

### 3.4 Amino acid contents

As can be seen in Table 6, with increasing addition of *L. ruthenicum* branches, the GABA content showed a tendency to increase and then decrease (*p* < 0.01). The H2 group was extremely significantly higher (*p* < 0.01) than theH1, H2, CON groups, respectively, with increases of 81.46, 53.47, 84.26%. The other indices were not significantly different (*p* > 0.05).

**Table 6:** Effect of *L. ruthenicum* branches on amino acid content of meat (%).

Items	Groups				SE	*p-*value		
	CON	H1	H2	H3		Total	Linear	Twice
Gly	58.06	72.39	64.58	66.61	2.635	0.425	0.454	0.278
Ala	253.20	253.19	253.19	253.19	13.95	0.749	0.557	0.361
GABA	3.24^Bb^	3.29^Bb^	5.97^Aa^	3.89^Bb^	0.318	<0.001	0.011	0.015
Ser	69.62	88.66	62.86	67.63	5.394	0.503	0.520	0.539
Pro	29.87	42.95	28.87	36.95	2.665	0.294	0.757	0.180
Val	45.93	69.17	52.51	50.94	4.348	0.428	0.966	0.190
Thr	36.64	57.93	42.16	38.021	3.784	0.305	0.724	0.117
Ile	37.35	48.66	34.25	36.25	2.909	0.451	0.502	0.454
Leu	87.33	112.25	77.89	84.35	6.694	0.435	0.475	0.517
Asn	43.51	52.25	51.47	47.30	2.771	0.741	0.686	0.304
Orn	15.72	12.97	20.63	13.34	2.064	0.549	0.979	0.611
Asp	28.07	21.95	31.24	26.38	2.415	0.675	0.853	0.905
Gln	271.10	264.35	331.79	304.96	18.752	0.625	0.339	0.807
Lys	68.18	89.64	67.59	74.75	6.919	0.776	0.971	0.645
Glu	83.31	71.73	78.68	59.33	6.534	0.569	0.289	0.784
Met	34.53	44.26	31.66	33.26	2.387	0.382	0.442	0.420
His	110.07	115.00	98.23	110.19	6.495	0.857	0.792	0.811
Phe	47.71	59.63	45.37	46.25	3.353	0.563	0.548	0.451
Arg	63.46	81.23	69.58	71.19	4.310	0.688	0.775	0.401
Tyr	45.11	57.18	29.57	36.27	4.301	0.182	0.146	0.751
Trp	11.03	13.55	10.51	9.96	0.908	0.646	0.464	0.443
DAA	438.08	495.97	487.36	454.71	18.788	0.744	0.816	0.288
EAA	368.70	495.10	361.96	373.77	29.555	0.498	0.661	0.371
NEAA	1469.56	1626.12	1529.72	1487.54	51.231	0.851	0.931	0.934
TAA	1443.05	1678.62	1528.07	1494.87	63.619	0.739	0.993	0.349

For the same row of data, there is no significant difference between groups without letters or with the same letters (*p* > 0.05), while different uppercase/lowercase letters indicate extremely significant/significant differences (*p* < 0.01/*p* < 0.05). CON: Control group; H1:10% *L. ruthenicum* branches; H2:20% *L. ruthenicum* branches; H3: =30% *L. ruthenicum* branches. EAA, Total essential amino acids. NEAA, Total non-essential amino acids. DAA, Total of major fresh-tasting amino acids. ΣTAA = *Sum of all amino acids in the table.* ΣEAA = Val + Leu + Ile + Phe + Ser + Lys + Met+Thr. ΣNEAA = Gly + Ala+Tyr + Asn + Glu + Cys + Pro+His+Arg.

ΣDAA = Glu + Ala+Arg + Gly.

### 3.5 Fatty acid content

As can be seen in Table 7, the increase in *L. ruthenicum* branches added to feed resulted in a trend of decreasing and then increasing SFA content. The H1 and H2 groups were significantly lower than the control group, decreasing by 6.87 and 7.07%, respectively (*p* < 0.05). The c20:0, c20:1 content showed a linear increase with increasing addition of black wolfberry branches, but the difference was not significant (*p* > 0.05).

**Table 7:** Effect of *L. ruthenicum* branches on fatty acid content (%).

Items	Groups				SEM	*p-*value		
	CON	H1	H2	H3		Total	Linear	Twice
c10:0	0.28	0.16	0.16	0.22	0.024	0.203	0.434	0.051
c12:0	0.34	0.18	0.21	0.39	0.050	0.422	0.715	0.112
c14:0	4.25	3.08	2.98	4.27	0.313	0.289	0.988	0.060
c14:1	0.11	0.34	0.35	0.28	0.050	0.293	0.255	0.129
c15:0	0.48	0.36	0.39	0.57	0.034	0.105	0.299	0.025
c16:0	26.81	26.82	26.51	26.88	0.235	0.955	0.970	0.728
c16:1	1.48	2.42	2.50	2.08	0.212	0.326	0.324	0.122
c17:0	1.32	1.10	1.15	1.34	0.052	0.275	0.802	0.059
c18:0	22.11	20.31	20.50	21.13	0.603	0.750	0.635	0.354
c18:1	37.65	38.95	38.38	35.02	0.694	0.196	0.168	0.095
c18:2	4.08	3.09	3.36	3.23	0.228	0.455	0.284	0.367
c20:0	0.12	0.11	0.13	0.17	0.008	0.051	0.022	0.097
c20:1	0.06	0.12	0.13	0.10	0.010	0.068	0.188	0.020
c18:3	0.71	0.24	0.28	0.56	0.100	0.296	0.634	0.073
c20:4	0.21	0.19	0.17	0.19	0.022	0.962	0.751	0.687
SFA	55.70^a^	52.12^b^	52.02^b^	54.97^ab^	0.612	0.046	0.631	0.007
UFA	44.30	45.35	45.18	41.45	0.722	0.197	0.173	0.099
PUFA	4.99	3.52	3.81	3.98	0.256	0.197	0.221	0.110
MUFA	39.30	41.84	41.37	37.48	0.894	0.301	0.457	0.084
PUFA/SFA	0.08	0.07	0.07	0.07	0.004	0.266	0.206	0.197

For the same row of data, there is no significant difference between groups without letters or with the same letters (*p* > 0.05), while different uppercase/lowercase letters indicate extremely significant/significant differences (*p* < 0.01/*p* < 0.05). CON: Control group; H1:10% *L. ruthenicum* branches; H2:20% *L. ruthenicum* branches; H3: =30% *L. ruthenicum* branches. SFA = saturated fatty acid, UFA = unsaturated fatty acid, MUFA = monounsaturated fatty acid, PUFA = polyunsaturated fatty acid.

### 3.6 Relationship between meat quality and content of amino acids and fatty acids

**Figure 2:**
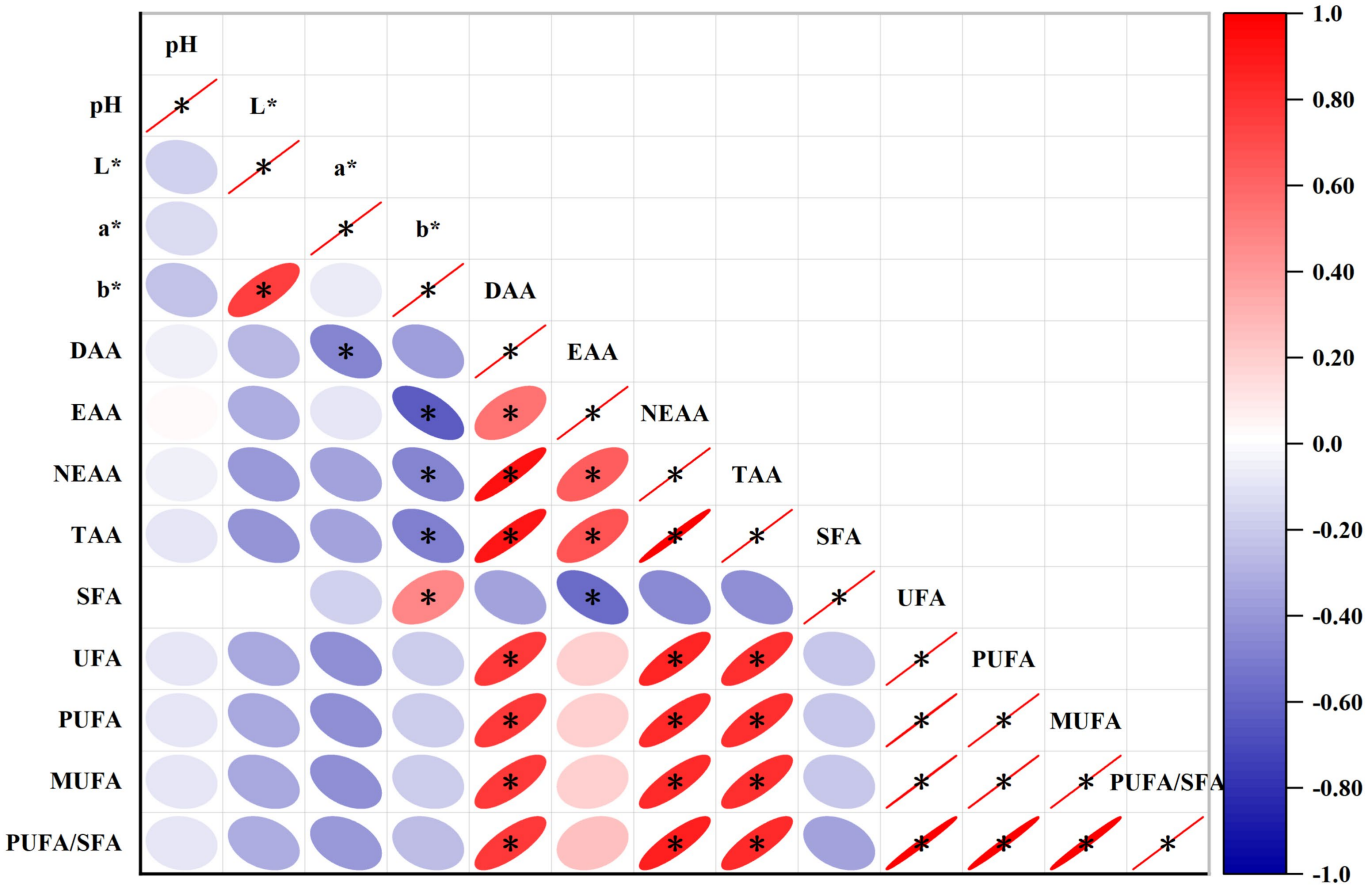
Heat map showing correlation between muscle amino acids, fatty acids and meat quality indicators. * represents 0.01 < *p* ≤ 0.05, and ** represents *p* ≤ 0.01.

As shown in Figure 2, b* was significantly positively correlated with L* and SFA, and significantly negatively correlated with EAA, NEAA, and TAA (*p* < 0.05). DAA was significantly negatively correlated with a*, and significantly positively correlated with, EAA, NEAA, TAA, MUFA, UFA, PUFA, and PUFA/SFA (*p* < 0.05). EAA was significantly positively correlated with NEAA, TAA and significantly negatively correlated with SFA (*p* < 0.05). NEAA was significantly positively correlated with TAA, UFA, PUFA, PUFA/SFA (*p* < 0.05). TAA was significantly positively correlated with UFA, PUFA, PUFA/SFA (*p* < 0.05). UFA was significantly positively correlated with PUFA, PUFA/SFA (*p* < 0.05). PUFA was significantly positively correlated with PUFA/SFA (*p* < 0.05).

### 3.7 Economic benefits

As can be seen in Table 8, the unit price for feed in each group ranged from 2.25–2.39 RMB/kg, and gradually decreased with increasing addition of *L. ruthenicum* branches. The gross profit of group H3 increased by 79.4% compared to CON.

**Table 8:** Effect of *L. ruthenicum* branches on economic benefits.

Items	Group				SEM	*p*-value		
	CON	H1	H2	H3		Total	Linear	Twice
Feed Unit price, RMB/kg	2.39^a^	2.36^b^	2.30^b^	2.25^b^	0.009	<0.001	<0.001	<0.001
ADFI, kg	1.78	1.69	1.78	1.82	0.035	0.624	0.995	0.207
Feed costs	257.12	246.12	242.73	222.32	2.248	<0.001	<0.001	<0.001
Sheep live weight unit price	31.00	31.00	31.00	31.00	–	–	–	–
Total weight gain, kg	11.91	11.89	12.43	14.15	0.533	0.172	0.065	0.225
Gain from weight gain	369.21	368.59	385.33	438.65	1.895	0.635	0.734	0.702
Net profit	114.08^b^	129.41^b^	139.99^b^	193.48^a^	1.932	0.038	0.008	0.963

For the same row of data, there is no significant difference between groups without letters or with the same letters (*p* > 0.05), while different uppercase/lowercase letters indicate extremely significant/significant differences (*p* < 0.01/*p* < 0.05). CON:Control group; H1:10% *L. ruthenicum* branches; H2:20% *L. ruthenicum* branches; H3: =30% *L. ruthenicum* branches.

## 4 Discussion

Feed intake, average daily gain, feed-to-weight ratio and final body weight are key indicators of growth performance. Among them, feed intake is significantly correlated with feed conversion efficiency (FCE), which is closely related to growth performance (16). The results of this study showed that the addition of up to 30% *L. ruthenicum* branch roughage to a standard diet could significantly increase the final weight of sheep. This was consistent with the findings of Duan et al. (12). Reflecting the fact that roughage made from *L.ruthenicum* branches is rich in polysaccharides, flavonoids, polyphenols and other biologically active substances, which could improve the growth performance of sheep by enhancing the digestion and absorption of nutrients. Through the analysis of economic benefits, H2 weight gain and economic returns were higher than the control group indicating that the LBP branch all-mixed pellet diet can improve the economic benefits of meat goats, can be applied in the practical production of meat goats and has a certain value of popularization.

The pH of muscle can reflect the speed and intensity of muscle glycolysis after slaughter, which affects its water holding capacity and color (17). Higher pH significantly improves the storage stability of meat by inhibiting the rate of glycolysis of myoglycogen, reducing water mobility, and maintaining protein conformational stability (18). As a reflection of muscle physiology and biology, meat color is an important indicator of appearance and economic value. Good color and luster can stimulate consumers’ desire to buy (19). Meat color (the a* value) is mainly influenced by hemoglobin and myoglobin, which turns bright red when oxygenated. The value of b* is affected by the intake of carotenoids in the rations as well as the inter- and intramuscular fat content. An increase in a* corresponds to a brighter red color, while a decrease in b* (yellow color) makes the meat appear more reddish, improving its sensory qualities and consumer appea (20).

It has been previously demonstrated that the addition of *Lycium barbarum* polysaccharides (LBPs) in the diet can enhance the appearance and sensory qualities of meat. Addition of LBPs improved meat quality by increasing the tethering force of the longest back muscles of lambs, increasing a* and decreasing b*; thus, it is reasonable to suppose that the polysaccharides in *L. ruthenicum* branch roughage could also have strong antioxidant functions, which could reduce glycogen fermentation and the accumulation of lactic acid in muscle, preventing the drop in pH that negatively affects quality (21). In this study, the value of L*, a* in the H2 and H3 groups was also significantly higher than that in the CON group. This suggests that the bioactive components of *L. ruthenicum* branches can prevent myoglobin oxidation and that phenols in flavonoid compounds with hydroxyl and carbonyl groups can bind metal ions and block the production of free radicals, thus enhancing the activity of iron myoglobin reductase and delaying the oxidation of myoglobin (22). Therefore, the improvement of meat quality by *L. ruthenicum* may be related to its strong antioxidant properties.

Certain amino acids (AAs), especially the four fresh-flavor AAs (Glu, Ala, Arg, Gly), are important flavoring substances, and the amino acid content in lamb directly affects its appeal to consumers (23). Studies have shown that the higher the content of flavor-enhancing AAs, the more delicious the meat (24). Currently, there are few reports on the effect of *L. ruthenicum* branches on the amino acid content of animal muscle. In this study, the addition of *L. ruthenicum* branch roughage improved the fresh-flavor AAs, but the difference was not significant. However, the *L. ruthenicum*-supplemented feed did significantly improve the nutritional value of the meat and this may be related to the fact that *L. ruthenicum* branches are rich in digestible protein and have a high EAA content. *L. ruthenicum* also provides beneficial gut bacteria to improve the gut microbial composition, which promotes the deposition of amino acids in the muscle (25). *L. ruthenicum* branches are also rich in flavonoids, as well as dietary fiber and active enzymes (26). GABA is effective in promoting protein metabolism, growth hormone mediator production, chondrocyte division and matrix proliferation, and in promoting glucose uptake and the synthesis of proteins in muscle (27). The addition of 20% of *L. ruthenicum* branches significantly increased GABA content in this study, which may be a result of its high flavonoid content. The strong antioxidant properties of flavonoids inhibit lipid oxidation and free radical generation in lamb, thereby protecting the activity of glutamic acid decarboxylase (GAD), a key enzyme that catalyzes the conversion of glutamate to GABA and whose activity is significantly affected by oxidative stress. Studies have shown that flavonoids can maintain the structural stability of GAD and promote the efficiency of GABA synthesis by scavenging reactive oxygen species (ROS) and reducing malondialdehyde (MDA) levels (28).

The most fatty acids in lamb meat are not only an important factor affecting the flavor, but also play a crucial role in human health and physiological well-being (29). Intramuscular fat content is one of the most important indicators for assessing meat quality and is closely associated with tenderness, flavor, and juiciness. It has been shown that flavonoids can significantly increase the fat content of the longest dorsal muscle of sheep, affecting the flavor of the meat (30). It was found that excessive SFA content in ingested meat increases the probability of coronary heart disease and can also lead to fat storage and inflammation in the human body, whereas in the present study, a significant decrease in the content of SFA in the longest muscle of the dorsum was found, which suggests that the extract of *L. nigra* is effective in improving the composition of the fat and decreasing the proportion of SFA, which has a positive impact on the improvement of the nutritional and health attributes of the meat.

The longest muscle of the back is very active in fatty acid oxidative metabolism and is dependent on fatty acids for energy, and the decrease in saturated fatty acid content of the longest muscle of the sheep’s back may be related to the flavonoid content in the branches of *L. ruthenicum* (31). Kwiecien et al. found that the bioactive components in alfalfa could increase the activity of SFA enzymes and promote the conversion of SFA to UFA (32). *L. ruthenicum* branches may also have the potential to improve the conversion and distribution of FAs in the dorsal muscle of sheep and have a positive effect on the nutritional value and flavor of the lamb meat. The potent active components (flavonoids, polysaccharides and polyphenols) in *L. ruthenicum* have antioxidant activities and reduce rumen microbial hydrogenation, leading to higher deposition of MUFA and PUFA in the muscles of meat sheep (33). Secondly, the high content of polyphenols in *L. ruthenicum* is also likely to be responsible for the improvement in fatty acid content. High doses of polyphenols may maintain the stability of polyunsaturated fatty acids in the meat by inhibiting lipid peroxidation and protecting the integrity of the muscle cell membranes, while promoting the efficiency of energy metabolism and significantly enhancing the end weight of sheep (Table 3) (1).

## 5 Conclusion

The results of this study show that *Lycium ruthenicum* branches can improve the growth performance of sheep and positively affect the amino acid and fatty acid composition of sheep muscle, thus improving meat quality and increasing profits for sheep farmers. Our data show that the optimal amount of *Lycium ruthenicum* branches for feed supplementation to achieve this goal is 20%.

## Data Availability

The original contributions presented in the study are included in the article/supplementary material, further inquiries can be directed to the corresponding author.
